# Contribution of the Microenvironmental Niche to Glioblastoma Heterogeneity

**DOI:** 10.1155/2017/9634172

**Published:** 2017-05-28

**Authors:** Ivy A. W. Ho, Winston S. N. Shim

**Affiliations:** ^1^Molecular Neurotherapeutics Laboratory, National Neuroscience Institute, Singapore; ^2^Department of Physiology, National University of Singapore, Singapore; ^3^National Heart Research Institute Singapore, National Heart Centre Singapore, Singapore; ^4^Cardiovascular and Metabolic Disorders Program, Duke-NUS Medical School, Singapore

## Abstract

Glioblastoma is the most aggressive cancer of the brain. The dismal prognosis is largely attributed to the heterogeneous nature of the tumor, which in addition to intrinsic molecular and genetic changes is also influenced by the microenvironmental niche in which the glioma cells reside. The cancer stem cells (CSCs) hypothesis suggests that all cancers arise from CSCs that possess the ability to self-renew and initiate tumor formation. CSCs reside in specialized niches where interaction with the microenvironment regulates their stem cell behavior. The reciprocal interaction between glioma stem cells (GSCs) and cells from the microenvironment, such as endothelial cells, immune cells, and other parenchymal cells, may also promote angiogenesis, invasion, proliferation, and stemness of the GSCs and be likely to have an underappreciated role in their responsiveness to therapy. This crosstalk may also promote molecular transition of GSCs. Hence the inherent plasticity of GSCs can be seen as an adaptive response, changing according to the signaling cue from the niche. Given the association of GSCs with tumor recurrence and treatment sensitivity, understanding this bidirectional crosstalk between GSCs and its niche may provide a framework to identify more effective therapeutic targets and improve treatment outcome.

## 1. Introduction

Glioblastoma (GBM), World Health Organization (WHO) grade IV glioma, is the most aggressive primary brain tumor in adults and accounts for over 50% of the tumors of the brain [1]. Current standard therapy after initial diagnosis includes maximal surgical debulking followed by adjuvant temozolomide (TMZ) administration and radiation therapy [2, 3]. Unfortunately, recurrent cases of GBM that are highly resistant to radiation and chemotherapy are common and relapsed patients have a dismal survival of less than 15 months [1]. These recurring malignant gliomas are highly infiltrative and may stem from a subpopulation of glioma stem cells (GSCs) that shares some characteristics with neural stem and precursor cells [4–10], such as self-renewal capability.

Two hypotheses have been proposed on the origin of such tumor heterogeneity. Clonal evolution hypothesis suggests that most cancers arise from a single altered cell which facilitates tumor initiation and progression. As the tumor progresses, accumulated genomic instability results in the appearance of new genetic variants. Those variants with selective growth advantage expand to become the predominant subpopulation in the tumor. The presence of multiple subpopulations in a tumor thus supports the theory of tumor heterogeneity. On the other hand, cancer stem cells (CSCs) hypothesis suggests that intratumor heterogeneity arises from CSCs that possess the ability to self-renew and initiate tumor formation. CSCs give rise to phenotypically diverse cancer cells and reside in specialized niches where interaction with the microenvironment regulates their stem cell behavior. This behavior suggests a possible linkage between therapy outcome and genomic composition of the tumor. Recent experimental evidence supports the concept of CSCs plasticity and the ability of non-CSCs to dedifferentiate into CSCs [11]. This concept is further supported by lineage tracing and clonal analysis experiments that demonstrate the hierarchical organization of tumor in vivo [12–14].

## 2. Molecular Heterogeneity of GBM

Advances in genomic sequencing and transcriptomic profiling reveal the existence of multiple molecular subtypes, namely, proneural, neural, classical, and mesenchymal, within a tumor, highlighting the heterogeneous nature of GBM [15, 16]. Each subtype is characterized by different transcriptional profile [15–17] and varied response to radiotherapy and chemotherapy [18–25]. The proneural GBM subtype can be further characterized as either isocitrate dehydrogenase-1 (IDH-1) wildtype or mutant. Mutation in IDH-1 results in remodeling of the glioma methylome, thus resulting in activation of gene expression characteristics of glioma CpG island methylator phenotype- (G-CIMP-) positive low grade tumor. Mutant IDH-1, which is G-CIMP-positive, has better prognosis and treatment response that is commonly seen in grade 2 and 3 tumor, thus representing secondary GBM [26, 27]. On the other hand, wildtype IDH-1, which is G-CIMP-negative, is characteristic of primary GBM that is more aggressive and less responsive to treatment than mutant IDH-1 [28]. The G-CIMP-negative GBM (IDH wildtype proneural, neural, classical, and mesenchymal) responds differently to standard therapeutic modality of temozolomide and radiation. IDH-1 wildtype proneural tumor is more amenable to standard treatment regimen than those presented with mesenchymal tumor subtype [18, 21, 29].

The existence of different molecular subtypes within a tumor [30] and at single cell level [31, 32] was demonstrated using genome wide gene expression analysis. Using fluorescence-guided multiple sampling approach, Sottoriva and colleagues showed that GBM tumor fragments harvested from spatially distinct location within the tumor can be classified into different molecular subtypes based on their gene expression profile [30]. Patel and colleagues revealed that all GBM contain heterogeneous mixtures of tumor cells using single cell transcriptomic analysis on 430 cells harvested from five GBM patients. They demonstrated that, regardless of the dominant subtype of the tumor, all tumors contain some cells having molecular characteristics that conform to the proneural subtype according to the Cancer Genome Atlas (TCGA) classification scheme [31], supporting the notion that all GBM subclasses evolve from the proneural subclasses [33]. Importantly, the group demonstrated that increased heterogeneity of the tumor correlates with poorer survival [31]. Using large-scale clonal analysis of glioma-initiating cells harvested from primary GBM, Segerman et al. further revealed the widespread and extensive heterogeneity that correspond to response to radiation and chemotherapy [32]. Resistant clones were associated with the mesenchymal cell state, which is consistent with previous reports in GBM and other carcinomas in which mesenchymal phenotype is associated with increased therapeutic resistance [34–36]. Given that all tumors contain a portion of proneural cells regardless of their dominant subclasses [31], it is conceivable that the clinical outcome of GBM tumor is greatly influenced by subtype of cells that coexist within that tumor environment.

It is now established that an epithelial-mesenchymal-like transition (EMT), termed as proneural-mesenchymal transition (PMT), exists in GBM [16, 35, 37]. Analysis performed on paired GBM specimens prior to radiotherapy and at the time of recurrence suggested that there is a shift of the glioma cells from the proneural to the mesenchymal phenotype [16]. In addition, transcription factors that play important role in EMT, such as twist family BHLH transcription factor-1 (TWIST-1), zinc finger E-box binding homeobox-1 (ZEB-1)/ZEB-2, and snail family transcriptional repressor-1 (SNAIL-1)/SNAIL-2, were found to be altered in GBM [38, 39] as the proneural cells undergo transformation to mesenchymal subtype. Downregulation of proneural-specific markers and upregulation of mesenchymal-specific markers were also observed in irradiated proneural glioma cells [35]. Furthermore, elevated levels of mesenchymal markers expression were observed in mouse xenograft model treated with bevacizumab, a monoclonal antibody against vascular endothelial growth factor (VEGF) [37]. The shift from proneural to mesenchymal phenotype may account for the enhanced aggressiveness observed in patients with recurrent glioma that have acquired resistance to bevacizumab [37]. This evidence collectively points to an intimate involvement of microenvironmental flow of signals in contributing to PMT, suggesting that targeting the microenvironmental niche is critical for controlling GBM.

## 3. Microenvironment and Tumor Heterogeneity

Heterogeneity among tumor cells not only arises within a single tumor as a result of molecular and genetic changes but also is affected by different microenvironments within different regions of the tumor [40–42]. GBM are highly vascularized, and often GSCs that are in the perivascular niches are observed to interact with endothelial cells (ECs) [43, 44]. Interactions between GSCs and their environment through autocrine or paracrine factors promote invasion and growth of GSCs and likely affect their response to therapy [45]. Likewise, GSCs exist in a particular niche will cross-influence the stemness of other GSCs. Understanding this bidirectional crosstalk between GSCs and its niches is critical to deciphering the regulatory role of the microenvironment on GSCs tumor initiation, invasion, therapeutic resistance, and heterogeneity.

### 3.1. Perivascular Environment

The perivascular niche of brain tumor is critical to the maintenance of CSC state of the tumor. GSCs are often found to adhere to vascular structures where physical interaction with ECs occurs [44, 46, 47]. The physical proximity of GSCs to ECs is a key driver of tumor progression [44] and interaction between tumor cells and ECs/pericytes has been reported to influence GBM malignancy [46]. ECs promote GSCs survival through secretion of soluble factors such as transforming growth factor-*β* (TGF-*β*) and platelet derived growth factor (PDGF), which in turn increase expression of stemness-related genes such as SRY-box-2 (Sox-2), oligodendrocyte lineage transcription factor-2 (Olig-2), Bmi-1, and CD133 in GSCs [4]. On the other hand, GSCs promote ECs angiogenesis through expression of proangiogenic factors such as VEGF.

Direct interaction between ECs and GSCs also activates key stemness pathways such as nitric oxide- (NO-) cyclic GMP pathway [48] and Notch signaling [49]. In the brain tumor microenvironment, NO is synthesized by nitric oxide synthase (NOS). There are three isoforms of NOS, neuronal NOS (NOS-1 or nNOS), inducible NOS (NOS-2 or iNOS), and endothelial NOS (NOS-3 or eNOS). nNOS and eNOS, which are constitutive and calcium-dependent, produce small amount of NO for very short period of time when activated. By contrast, iNOS is calcium-independent and generates high concentration of NO that last for longer intervals when activated. All three NOS isoforms are highly expressed in high grade glioma in comparison to the lower grade tumors [50–54], with the exception of iNOS, which is also highly expressed in GSCs [55]. The cell autonomous increase in iNOS expression in GSCs results in enhanced neurosphere formation and tumorigenic potential and correlates with poor patient survival [55]. Interestingly, NO production in the tumor microenvironment may also be regulated by interaction between the glioma cells and ECs. This reciprocal production of NO by the glioma cells (nNOS) and ECs (eNOS) may represent another way of direct crosstalk between cells in the microenvironment that facilitate tumorigenesis [56, 57]. iNOS-induced tumorigenesis can be abrogated by the expression of the NO consuming enzyme flavohemoglobin [55]. Indeed, when iNOS was targeted using either small molecular inhibitor or shRNA, there was a significant loss in tumorigenesis in both human and murine glioma cells [55], demonstrating that GSCs-derived iNOS may be a potential therapeutic target. In addition to NO signaling, Notch signaling between cells of the microenvironment also promotes GSCs-mediated tumorigenesis. Notch-1 and Notch-2 are expressed on GSCs, while their ligands, Delta-like-4 (DL-4) and Jagged-1, are expressed on the ECs [49]. Abrogating Notch signaling through targeted knockdown of DL-4 or Jagged-1 in brain microvascular endothelial cells has been shown to reduce tumor angiogenesis and tumor growth [49]. On the other hand, using a PDGF-driven glioma model, Charles and colleagues found that eNOS maintain GSCs phenotype and enhanced tumorigenesis via activation of Notch signaling [56]. However, Notch signaling was suppressed when eNOS expression was blocked and thus reduced tumor growth and prolonged survival of tumor bearing mice [56]. In addition to Notch and NO, PDGF also induces stemness associated genes in patient-derived neurosphere lines via inhibitor of differentiation (ID) [58], which functions to maintain GBM mesenchymal subclass and promote adherence of GSCs to the perivascular niche [59]. Inactivation of ID protein resulted in loss of GSCs contact with the ECs and loss of self-renewal [60]. Thus, it appears that ID, NO, PDGF, and Notch individually enhance the self-renewal capacity of GSCs. But together, the PDGF-ID-NO-Notch signaling axes not only maintain and promote GSCs phenotype but also enhance tumor angiogenesis [61].

GSCs are not passive recipient in the microenvironment; in fact, they play active participatory role such as stimulating angiogenesis through expression of angiogenic factors such as VEGF [43, 62]. Recent studies have suggested that GSCs may differentiate into ECs [63, 64] and pericytes [14]. Ricci-Vitiani and coworkers found that a percentage of ECs within GBM contain somatic mutations identical to the tumor cells, suggesting neoplastic origin of the vascular endothelium [63, 64]. However, large-scale analysis of patient-derived brain tumors suggested that tumor-derived endothelial cells are rare events [65]. By contrast, GSCs differentiation into pericytes was shown to promote vessel maturation. Using lineage tracing of GSCs from 21 GBM xenografts, Cheng and colleagues demonstrated that GSCs give rise to pericytes in vivo in part through EC-derived TGF-*β* signaling [14]. Importantly, tumor vessels with few pericytes coverage appeared to be more sensitive to radiation and chemotherapy treatment [66, 67]. On the other hand, high pericyte coverage stabilizes vessels and promotes perfusion and thus promotes tumor growth [68]. The results from Cheng et al.'s studies further demonstrated that depletion of GSCs-derived pericytes results in inhibition of tumor growth, thus suggesting a possible utility of targeting GSCs-derived pericytes for treating GBM.

GSCs not only interact with ECs in the perivascular niche but also interact with the extracellular matrix (ECM). Abnormal ECM remodeling affects ECs, immune cells, and tumor angiogenesis, which influences GBM progression and invasion. ECM components, such as laminin, integrin, vitronectin, and fibronectin, have been shown to associate with tumor grade and patient survival. The laminin family of proteins consists of five laminin *α* chains, four laminins *β* chains, and three laminins *γ* chains. These *α*, *β*, and *γ* subunits form heterotrimers to promote downstream signaling including promotion of cell adhesion and migration, regulation of cell proliferation, differentiation, and survival [69, 70]. Among them, *α*2 and *α*4 laminins are primarily expressed in mesenchymal cells. Specifically, laminin *α*2 expression is higher in classical and mesenchymal subtypes than neural and proneural subtypes and correlates with poorer survival. Lathia et al. demonstrated that laminin *α*2, which is expressed in non-GSCs and ECs, plays a role in GSCs maintenance. Targeted knockdown of laminin *α*2 using shRNAs decreased the clonogenic and proliferative capacity of GSCs. Further, depletion of laminin *α*2 results in increased tumor latency in mice [71]. Laminin *α*4 is also expressed in glioma and other tumor cells, especially after EMT, and contributes to tumor invasion and recurrence [72–78]. The laminin receptor, integrin *α*6*β*1, regulates tumor cells survival and promotes EC growth in GBM [79] and is required for GSCs maintenance [80, 81]. In the perivascular niche, integrins mediate the interaction between tumor cells and ECs, thus maintaining the function of the niche. Similar to laminin, alteration in integrin expression is associated with tumor malignancy [82]. In fact, prosurvival integrin-mediated signaling following radiation and chemotherapy occurs at the ECM. Integrins are heterodimeric cell adhesion molecules formed by dimerization of 18 *α*-subunits and 8 *β*-subunits. Integrins *α*v*β*3, *α*v*β*5, *α*5*β*1, *α*3, and *α*6 are expressed in GBM [83–86]. In particular, *α*v*β*3 and *α*v*β*5 are enriched in highly vascularized GBM; as a result, targeting these integrins with cilengitide was evaluated in clinical trials. Unfortunately, results from recent CENTRIC trial did not demonstrate improved outcomes for patient treated with cilengitide in combination with temozolomide and chemoradiotherapy [83]. Despite the setback, integrin remains a potential target because GSCs also express high levels of *α*3 and *α*6 [85]. Overexpression of integrin *α*3 has been shown to promote GBM invasion via the ERK1/2 signaling in human astrocytoma and GBM patient-derived xenograft mouse model [84]. On the other hand, integrin *α*5*β*1, which is expressed in mesenchymal GBM and is associated with increased invasion, negatively regulates the p53 pathway to modulate prosurvival molecule survivin in GSCs and mouse xenograft model [86].

Another molecule that is highly expressed in the ECM at the perivascular niche is cadherins, which mediate cell-cell interactions in multiple processes including tumor invasion [87–89]. Cadherins mediate cell adhesion through interaction with *β*-catenin, protein kinase C, cdc42, and Numb. N-cadherin is expressed in normal stem cell niche and is required for maintenance of progenitor state [90]. On the other hand, E-cadherin is downregulated in GBM and is associated with poor prognosis [91]. Alteration in cadherin expression is found to associate with a change in tumor phenotype and growth. For example, inhibiting VEGF pathway induces a switch from angiogenic to invasive phenotype, which is followed by a switch in cadherin subtype. Specifically, cadherin 11 is highly expressed in mesenchymal GBM subclasses and is associated with enhanced GBM migration and tumor growth in vivo [92].

In summary, bidirectional crosstalk between GSCs and the microenvironment in the perivascular niche enhances stem cells phenotype of GSCs and promotes glioma cell invasion, proliferation, and resistance to therapy. GSCs promote ECs recruitment and induced expression of angiogenic factors to support angiogenesis. Thus, deciphering the molecular mechanisms involved in the interaction between GSCs and the perivascular environment could well reveal insights into the complicated nature of glioma tumorigenesis.

### 3.2. Hypoxic Environment

Hypoxia is a hallmark of GBM [45, 93–96]. Hypoxia stimulates the expression of the transcription factor hypoxia-inducible factors (HIF), which results in downstream activation of proangiogenesis factors such as angiopoietins, TGF-*β*, PDGF/PDGF-R, and VEGF/VEGF-R [97]. In addition to triggering multiple signaling pathways that affect GSCs self-renewal, proliferation, cell invasion, and survival [98], hypoxia also influences therapeutic resistance of GBM and enhances genetic instability of tumor cells. The low oxygen content in the tumor tissues not only attenuates expression of DNA mismatch repair genes but also inhibits free radicals generated from radiation treatment and thus impedes therapeutic efficacy and encourages rapid development of drug resistance phenomenon [99]. Furthermore, HIF-1 activates the multidrug resistance-1 (MDR-1) gene, which encodes P-glycoprotein (P-gp) ATP binding cassette transporters, in response to hypoxia. Activated P-gp acts as a drug efflux pump to remove intracellular concentration of chemotherapeutic drug and hence renders treatment ineffective.

GSCs are enriched in the hypoxic regions of GBM tumor and are characterized by reduced oxygen tension and activation of HIF-1 and HIF-2 [100]. HIF-1*α* is highly expressed in GBM in particular in hypoxic cells forming pseudopalisades around regions of necrosis and invading cells [101]. However, the degree of hypoxia differentially influences the expression of HIF-1*α* and HIF-2*α*. Severe hypoxic conditions result in upregulation of both HIFs in GSCs, while HIF-1*α* expression is also observed in nonstem cells and neural stem cells in addition to GSCs in mild hypoxia [99]. Both HIF-1*α* and HIF-2*α* are required for GSCs maintenance because targeted knockdown of either HIFs impaired GSCs self-renewal [47, 102]. Whereas HIF-2*α* promotes GSCs phenotype, HIF-1*α* is required for GSCs maintenance. HIF-2*α* upregulates a number of GSCs genes responsible for induction of a pluripotent state such as Kruppel-like factor-4 (Klf-4) [103], Sox-2, and Oct-4 [47, 104, 105]. Furthermore, HIF-2*α* also activates c-Myc [97, 98], a stem cells regulator, suggesting its role in regulating undifferentiated phenotype of CSCs in the hypoxic niche. Exposure to long-term hypoxia induced a phenotypic shift towards a stem-like state [106] that is accompanied by upregulation of Oct-4, Nanog, Sox-2, c-Myc, and nestin, all of which play roles in reprogramming [106–108]. In fact, this phenotype shift is observed in GBM patients who underwent bevacizumab treatment. Bevacizumab treatment, which targets VEGF, initially normalizes the vessels integrity and permeability [109]. However, prolong treatment of bevacizumab beyond the normalization phase induces hypoxia which recruits bone marrow-derived myeloid cells to glioma tissues [110, 111]. Furthermore, comparison of GBM patient tumor samples obtained before and after bevacizumab treatment showed increased intratumoral hypoxia [112] and upregulated level of c-Met expression [113]. Hypoxia has been shown to induce c-Met expression [114]. Along the same line, c-Met transcription is activated by HIF-1*α*, which results in enhanced cell invasion upon activation by hepatocyte growth factor (HGF) [115]. Thus, the upregulated level of c-Met observed in bevacizumab-treated tumor may be a response to hypoxia as a result of prolong anti-VEGF therapy. In contrast to this study, Bergers and team observed elevated concentration of phosphorylated c-Met expression at the invasive edge of mouse tumors that are not hypoxic, rather than at the tumor core which is hypoxic [116], suggesting that invasive phenotype is not solely driven by higher oxygen tension. Together, these findings illustrate the plasticity of the microenvironment in shaping tumor invasiveness through the same signaling pathway.

Hypoxic regions in GBM are spatially heterogeneous, with some regions having higher degree of severity than others, indicating that individual tumor cells may respond to a range of oxygen tension in the microenvironment. These localized hypoxic regions promote a malignant phenotype clinically and may contribute to the heterogeneity of the tumor [100]. The heterogeneous hypoxic zone also contributes to heterogeneity in metabolic reprogramming. Indeed, glycolytic enzymes and glucose transporters such as Glut-1 and Glut-3 as well as lactate exporters and pH regulators such as monocarboxylate transporters (MCTs) and carbonic anhydrases are induced by hypoxia [117–119]. HIFs also promote the expression of metabolites such as hexokinase, aldolase, and carbonic anhydrase which could in turn stimulate glycolytic flux and increase lactate buildup in the extracellular space [117, 118, 120]. Importantly, these metabolic enzymes and transcriptional regulators converge into multiple pathways that encourage tumor growth. The heterogeneous nature of the metabolic microenvironment can be driven by activation of phosphoinositide 3-kinase (PI3K)/Akt, Myc, or p53, which orchestrate glycolysis [121–123], glutaminolysis, and lipid synthesis [124, 125] pathways [126, 127].

GBM displays the Warburg effect, which is a preference to utilize aerobic glycolysis for energy instead of oxidative phosphorylation. Overexpression of metabolic enzymes such as hexokinase 2 (HK2) that is required for metabolic reprogramming has been shown in GBM, but not in low grade brain tumor [128]. In addition to pyruvate dehydrogenase kinase 1 or Glut1/4 [128–130], GBM also expresses UDP-Glc and UDP-glucuronic acid (UDP-GlcA) and crucial substrates of glycosaminoglycan (GAG) synthesis, during hypoxia [131]. Upregulation of UDP-GlcA suggests reshaping of the tumor microenvironment through altered GAG synthesis [132] during hypoxia. Notwithstanding, metabolic needs of tumor cells are dynamic and may depend on the severity of hypoxia [133–135], exposure to cytokines, or extracellular matrix proteins [136]. Metabolic symbiosis between hypoxic and aerobic tumor cells was recently reported in which metabolic substrates such as lactate produced in hypoxic cells are taken up by normoxic cancer cells and used as fuel [137–139]. This symbiotic microenvironment may promote subtype switching.

In GBM, IDH-1 wildtype proneural subclasses are characterized by having high glutamate level [140]. On the other hand, aldehyde dehydrogenase-1A3 (ALDH-1A3), an isozyme of ALDH-1 in the glycolysis and gluconeogenesis pathway, is elevated in mesenchymal subclasses [35] through the activation of the transcription factor FoxD1 [141]. Blocking ALDH-1 activity with inhibitor diethylaminobenzaldehyde (DEAB) or a novel class of imidazo [1, 2-*a*] pyridine derivatives reduced mesenchymal tumor cells growth and inhibited xenograft growth in glioma bearing mouse brains [35, 141]. Using a collection of seventeen patient-derived GSCs, Marziali and colleagues found that the proneural-like GSCs express metabolites such as N-acetylaspartate (NAA) and *γ*-aminobutyric acid (GABA), which is involved in the production of neurotransmitters. Conversely, mesenchymal-like GSCs lack NAA and GABA but have high levels of high mobile lipids indicative of an astroglial-like metabolism [142], thus demonstrating the differential metabolic programming in different molecular subclasses. Interestingly, Marin-Valencia and team suggested that GBM can utilize both glycolysis and mitochondrial glucose oxidation as their energy source as revealed by carbon-13 nuclear magnetic resonance spectroscopy of GBM xenograft in vivo [143]. Along the same vein, Janiszewska et al. found that the oncofetal insulin-like growth factor 2 mRNA-binding protein 2 (IMP2, IGFBP2), which plays a role in oxidative phosphorylation, is a key regulator in proneural GSCs and correlates with poor prognosis [144]. Unlike mesenchymal GSCs, inhibition of oxidative phosphorylation, but not glycolysis, abolishes GBM cells clonogenicity in proneural GSCs, suggesting that mesenchymal and proneural GSCs preferentially utilize different pathways for fuel [143]. How the metabolic pattern differs between different molecular subtypes remains unclear, but it is highly probable that intricate crosstalk between the microenvironment and the tumor may influence the metabolic profile of the tumor.

Several oncogene driven pathways converge to drive changes in metabolic programming. A key to understanding the therapeutic significance of metabolic changes in the tumor is to integrate information obtained through omics profiling via genomics, epigenetics, and transcriptomics as well as metabolomics of different stages of tumor, so as to decipher critical enzymatic players at a systems level for possible therapeutic targets.

### 3.3. Inflammatory Environment

Vascular abnormalities in GBM can result in disruption of the blood-brain barrier (BBB). BBB is composed of astrocytes, endothelial cells, and pericytes that tightly regulate the transfer of molecules between the blood and the brain. Intact BBB ensures that the brain is immune-privilege [145]. Disruption of the BBB, arising from either loss of vessels integrity or displacement of astrocytes by glioma cells, allows the influx of circulating immune cells. Monocytes [146], neutrophils [147], and myeloid-derived suppressor cells (MDSC) [148, 149] are commonly found within the tumor microenvironment [150–153]. These cells form another component of the heterogeneous tumor microenvironment, where crosstalk among the various members promote angiogenesis, convey immune-suppressive functions, and promote tumor growth and progression.

In the tumor microenvironment, tumor associated macrophages (TAMs) are commonly found in the vicinity of GSCs and correlate with the density of GSCs perhaps owing to the higher level of chemoattractants such as VEGF [146, 154]. The percentage of TAMs infiltration into a tumor is positively correlated with the tumor grade [155]. TAMs can be defined into either type 1 macrophages (M1)/Th1 (type 1 T helper cells) or type 2 macrophages (M)/Th2. Classically activated M1 macrophages stimulate antitumor response by production of proinflammatory cytokines, presenting antigens to adaptive immune cells and phagocytosing tumor cells. On the other hand, the alternatively activated M2 macrophages express immunosuppressive cytokines, intracellular signal transducer, and activator of transcription 3 (STAT3) and scavenger receptors such as CD163, CD204, and CD206 and promote tumor supportive CD4+ regulatory T cells [156–159]. GSCs secrete soluble factors, such as periostin, that recruit and support the growth of macrophages through integrin *α*v*β*3 [160]. Conversely, molecules produced by TAMs, such as TGF-*β*, stromal-derived factor-1 (SDF-1), and NO, maintain and promote GSCs [161–164]. TGF-*β* plays dual role in the tumor microenvironment. On the one hand, TGF-*β* released from TAMs induces matrix-metalloproteinase-2 (MMP-2) and MMP-9 expression from GBM to augment GC invasion [111, 165–167]. On the other hand, TGF-*β* released from GSCs actively suppresses M1 macrophages, inhibits phagocytosis, and induces polarization of microglial and macrophages into the immunosuppressive M2 phenotype and thus enhances the capacity of TAMs to inhibit T cell proliferation, thereby promoting tumor progression [164, 168].

MDSCs are a heterogeneous population of immature myeloid progenitors that mediate immune suppression and support glioma growth, invasion, vascularization, and expansion of regulatory T cells via various molecules. It is believed that MDSCs interact with gliomas and GSCs [169]; however, the exact mechanisms of crosstalk remain undefined. Using CD133 and Sox2, Otvos and colleagues found significant amount of MDSCs located directly adjacent to GSCs [149], suggesting possible interaction between GSCs and MDSCs in the tumor microenvironment. Although the mechanism of crosstalk is not clear, what we do know is that glioma cells recruit immature myeloid cells to promote their differentiation into MDSCs either through direct cell-cell contact or the release of soluble factors or exosomes. MDSCs primed with GSCs conditioned media were found to have elevated ratio of CD4-positive to CD8-positive T cells and decreased interferon-*γ* (IFN-*γ*) production, suggesting that soluble factors secreted by GSCs exerted an immunosuppressive phenotype in MDSCs [149]. Using a cytokine screen performed on GSCs and nonstem tumor cells conditioned media, Otvos et al. found significantly higher level of migratory inhibitory factor (MIF) in GSCs conditioned media. MIF regulate arginase-1 production through a C-X-C-chemokine receptor-2 (CXCR-2)-dependent manner [149]. Arginase-1 plays a role in MDSCs-induced immunosuppression by depleting L-arginine essential for growth and differentiation of T cells, resulting in T cell dysfunction [170]. The group further demonstrated that blocking MIF using shRNA reduced arginase-1 production. Furthermore, depleting MIF in immunocompetent mouse glioma using shRNAs increased tumor latency and proportion of cytotoxic T cells but decreased T_Regs_ [170]. Similarly, Domenis et al. also demonstrated that exosomes released by GSCs promoted immunosuppressive phenotype in monocytes and stimulated production of arginase-1 and interleukin-10 (IL-10) by monocytic-MDSCs [169]. Together, these studies demonstrated that MDSCs interact with GSCs to modulate glioma aggressiveness by immunosuppressing monocytes and other T cell populations.

It is interesting to note that each molecular subtype has different frequency and types of immune infiltrates. Higher frequency of TAMs was detected in mesenchymal subtypes in comparison to other GBM subtypes [171]. In fact mesenchymal subtype GBM is mainly infiltrated with microglia, whereas proneural and neural subtypes contain similar frequency of MDSCs, microglia, and macrophages, while classical subtype has a higher percentage of MDSCs [152]. However, implications of the different percentage and type of immune cells in response to therapy remain unknown. Interestingly, PMT signaling pathways are found to be upregulated in TAMs, indicating a role of the immune cells in influencing GBM heterogeneity [152, 172] and modulating mesenchymal differentiation [34].

Triggering PMT in tumor cells in the perivascular niche not only induces stem cells phenotypes but also maintains the stemness of cancer cells. One of the molecules that is crucial in PMT is osteopontin, which is secreted by immune cells under inflammatory conditions and promotes GSCs phenotype by activating CD44 [173]. Higher level of osteopontin expression is found in mesenchymal GBM when compared with other GBM subtypes. This finding is consistent with the preclinical finding that osteopontin expression is higher in murine microglia than in macrophages in GL261 mouse glioma model [174]. Whereas mesenchymal GBM also express CD44 at high level, CD44 expression in proneural tumors is confined to the perivascular niche [173]. Moreover, CD44 expression correlates with hypoxia-induced gene signatures and poor survival [173]. An elegant study by Bhat et al. showed that subtype switching from proneural to mesenchymal can be driven by paracrine factors such as tumor necrosis factor-*α* (TNF-*α*), which in turn activates transcription factor nuclear factor-*κ*B (NF-*κ*B) to drive mesenchymal transition [34]. Another study identified the transcriptional coactivator with PDZ-binding motif (TAZ) as a key mediator of mesenchymal phenotype in GBM [175]. Silencing of TAZ in mesenchymal GBM decreased expression of mesenchymal markers; on the other hand, overexpression of TAZ in proneural GSCs induced mesenchymal markers expression [175]. Together with STAT3 and CCAAT-enhancer-binding proteins-*β* (CEBP-*β*), CD44 and NF-*κ*B activation portends poor survival in GBM patients [34]. Importantly, these transcription factors also play important role in inflammatory response, further reinforcing the concept that crosstalk between paracrine factors and inflammation-associated transcription factors drives mesenchymal transition [34, 35, 176].

It remains unclear what factors are responsible for mediating the interaction between GSCs, ECs, and TAMs. Given the complexity of the glioma inflammatory niche, elucidating the molecular mechanism involved in various interactions is critical to address several key questions: do TAMs acquire dissimilar function when interacting with different microenvironment generated by the various molecular subclasses? Does combining TAMs targeted therapy with standard treatment regimen give better treatment response than GSCs-targeted therapy, irrespective of the molecular subclasses? More importantly, is subtype-specific therapy essential for personalized medicine? These are some of the questions that remain to be addressed; TAMs remain a promising target for the design of therapeutic intervention.

## 4. Concluding Remarks

**Figure 1:**
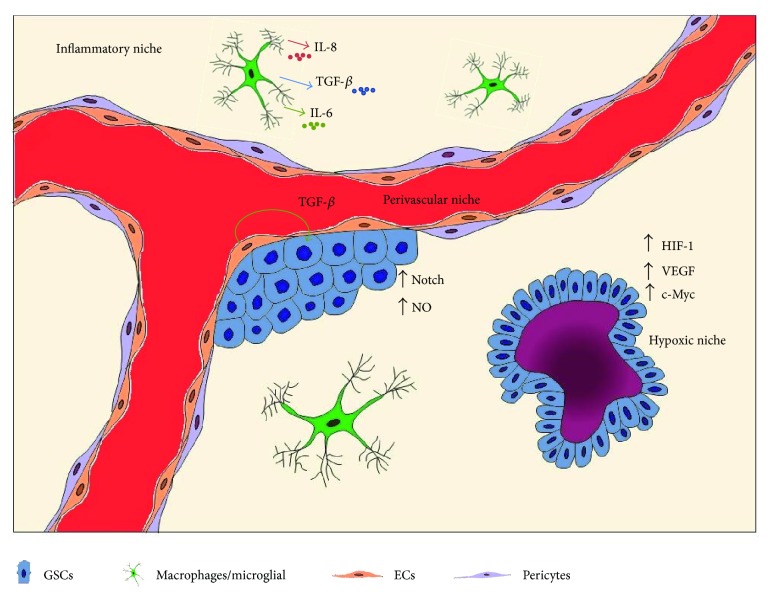
Tumor microenvironment and its effect on GSCs. Dialogues between tumor cells and other cell types in the microenvironment create vascular niches that regulate tumor growth. The perivascular niche contains cells such as ECs, pericytes, astrocytes, macrophages, and microglial. Each component of the perivascular niche interacts with GSCs to promote glioma cells growth and proliferation, maintain GSCs stemness, and control vascular integrity. GBM contains areas of pseudopalisading necrosis that is the core of the hypoxic niche. Hypoxia upregulates HIFs that induce the expression of oncogenes and transcription factors such as c-Myc and STAT3 involved in stem cells maintenance and expansion. Hypoxia also contributes to metabolic programming and recruitment of macrophages and microglial. These cells form the inflammatory niche, where TAMs secrete soluble factors such as TGF-*β* and IL-6 that expand GSCs population and promote glioma invasion. Interaction between GSCs and the various players in the microenvironment orchestrate tumor cells responds to therapeutic interventions, thus contributing to the heterogeneity of the tumor.

The inherent plasticity of GSCs thus suggests that its response to environmental cue is an adaptive response. Given the correlation of GSCs with tumor recurrence and response to treatment, further understating of GSCs biology and its surrounding niche may provide a framework to identify more effective treatment interventions (Figure 1).

Targeting the tumor microenvironment represents a promising approach to prevent tumor progression. However, several key questions remain unanswered. Although different molecular subtypes exhibit varying degree of hypoxia and immune cell infiltration, it is still not clear whether there are differences in the composition of the distinct tumor niches. Patient stratification for molecular therapies using tissues obtained from single surgical site may not present accurate information, as evidenced by the significant differences between samples obtained from different regions from the same tumor mass. Given that the tumor cells are genetically and epigenetically diverse, it is conceivable that the interaction between the tumor cells and the microenvironment is niche type specific to better accommodate the needs of the GSC. This heterogeneity in the niche will affect tumor cells response to therapies. Furthermore, the tumor microenvironment is dynamic. Changes in oxygenation, which in turn affects hypoxic conditions and metabolic states, neovascularization, and tumor invasiveness, will alter receptivity of the niches to accommodate more aggressive GBM growth. On the other hand, therapies may convert a tumor niche into a benign type or even eliminate it. For example, radiation therapy may convert the perivascular niche into hypoxic niche that promotes mesenchymal tumor growth. Conversely, short-term anti-VEGF therapy may convert a hypoxic niche into the perivascular niche that normalizes the vasculature and hence facilitates drug delivery. However, it is difficult to target specific niches for therapy; hence deciphering common shared pathways among various niches is the most efficient approach in designing therapeutic strategies. Thus, a more structured modulation of the glioma microenvironmental niche may complement the conventional treatments to achieve more effective control of the malignancy of GSC-driven GBM.
